# Genomic Analysis of a Novel Spontaneous Albino C57BL/6N Mouse Strain

**DOI:** 10.1002/dvg.22398

**Published:** 2013-04-26

**Authors:** Edward Ryder, Kim Wong, Diane Gleeson, Thomas M Keane, Debarati Sethi, Sapna Vyas, Hannah Wardle-Jones, James N Bussell, Richard Houghton, Jennifer Salisbury, Nina Harvey, David J Adams, Ramiro Ramirez-Solis

**Affiliations:** The Wellcome Trust Sanger InstituteHinxton, Cambridgeshire CB10 1SA, United Kingdom

**Keywords:** mouse, genome sequencing, genetics, transgenics, mammalian, genomics

## Abstract

Summary: We report an albino C57BL/6N mouse strain carrying a spontaneous mutation in the *tyrosinase* gene (C57BL/6N-*Tyr^cWTSI^*). Deep whole genome sequencing of founder mice revealed very little divergence from C57BL/6NJ and C57BL/6N (Taconic). This coisogenic strain will be of great utility for the International Mouse Phenotyping Consortium (IMPC), which uses the EUCOMM/KOMP targeted C57BL/6N ES cell resource, and other investigators wishing to work on a defined C57BL/6N background. genesis 51:523–528.

Members of the International Mouse Phenotyping Consortium (IMPC) (Brown and Moore, 2012) use mutant embryonic stem (ES) cells from the NIH’s Knockout Mouse Project (KOMP), the European Conditional Mouse Mutagenesis (EUCOMM) program, Regeneron, and The Canadian NorComm programme (Bradley *et al.,* 2012; Skarnes *et al.,* 2011) to generate mouse strains for high-throughput phenotyping, and for distribution to the research community. The majority of the ES cell resources used by the IMPC are made in JM8-derived cell lines, which were originally derived from C57BL/6N embryos (Pettitt *et al.,* 2009). To achieve the goal of producing and phenotyping mutant strains on an inbred genetic background, chimeras are bred to C57BL/6N (black) females. When an albino host blastocyst is used for chimera production this prohibits the assessment of germline transmission (GLT) using the coat color of G_1_ mice, and therefore all progeny of chimera matings must be biopsied and genotyped, with consequent ethical and cost/labor concerns. Although agouti or non-agouti host blastocysts can be used to aid subsequent color selection in certain combinations, their utility in high-throughput production is limited as they must be paired correctly with the agouti status of the injected ESC clone (Pettitt *et al.,* 2009).

During routine colony expansion a strain generated by the Sanger Mouse Genetics Project (MGP) (Colony ID: MDDH, EUCOMM ES cell clone EPD0176_3_A10 which carries the *Stard7^tm1a(EUCOMM)Wtsi^* allele) produced albino mice from the first intercross of germline animals. A new colony of these mice was established (Colony ID: MWTH) from offspring selected to be wildtype for the *Stard7^tm1a(EUCOMM)Wtsi^* allele so the causal mutation could be identified.

## RESULTS

**FIG 1:**
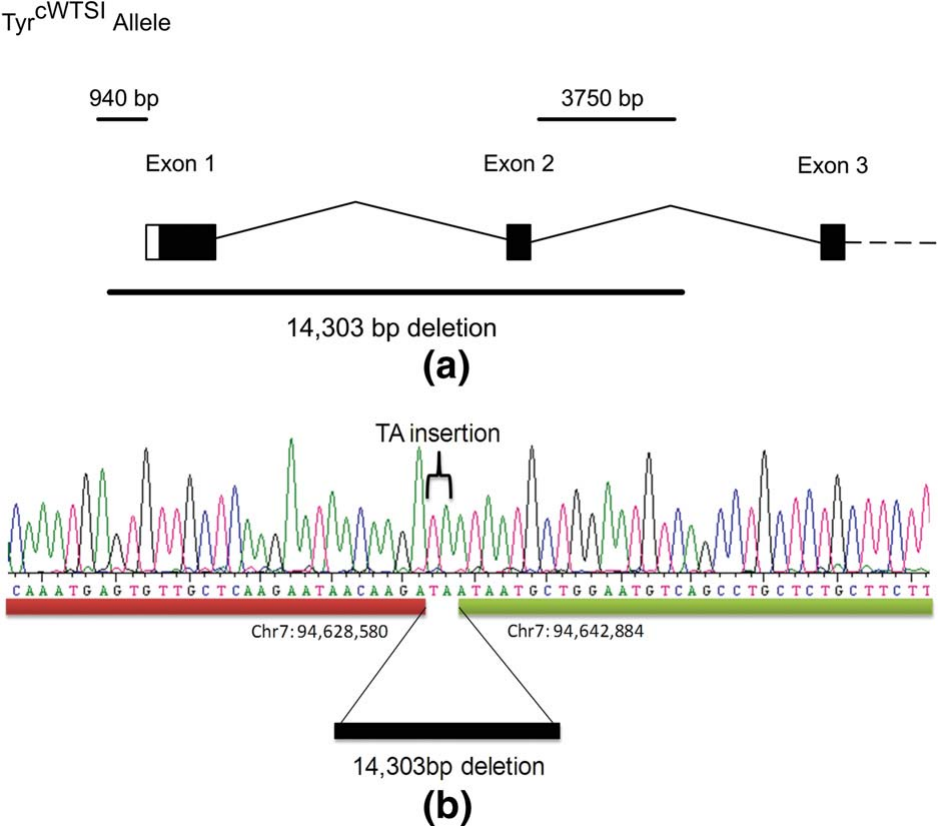
a: The Tyr^cWTSI^ deletion. The deletion spans from 940 bp 5′ to exon 1 from 3570 bp 3′ of exon 2 and completely removes exons 1 and 2. b: The sequence breakpoint of the Tyr^cWTSI^ deletion.

**FIG 2:**
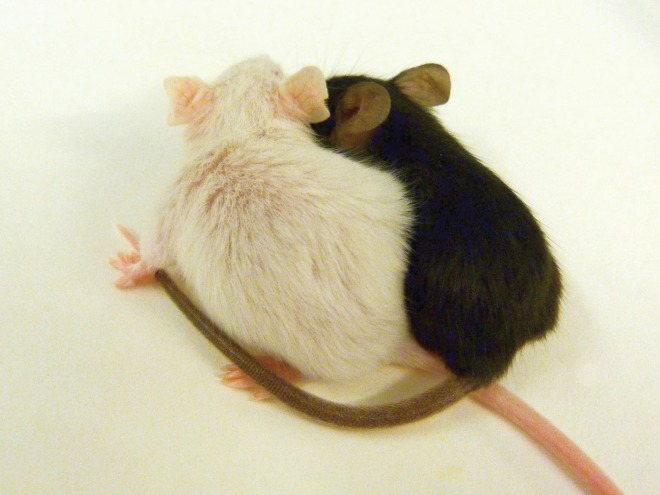
A C57BL/6N-Tyr^cWTSI^ mouse (left) compared to a black C57BL/6N mouse (right). Image taken at 4 weeks of age.

PCR analysis of the *tyrosinase* gene (the most likely candidate locus) failed to amplify exons 1 and 2 from albino mouse DNA. Further analysis of a 24 kb PCR tiling path using PCRTiler (Gervais *et al.,* 2010) revealed a 14.3 Kb deletion 940 bp 5′ of exon 1, including the minimal promoter region (Klüppel *et al.,* 1991), extending 3.75 kb 3′ of exon 2; in addition, a 2 bp TA insertion was found at the breakpoint (Fig 1). We refer to this new allele as *Tyr^cWTSI^,* and thus our albino strain is C57BL/6N-*Tyr^cWTSI^* (Fig 2). Analysis of DNA from the ES cell clone EPD0176_3_A10 revealed that the *Tyr^cWTSI^* mutation originated in this line, possibly during tissue culture. The mutation was not present in a random selection of 96 other EUCOMM/KOMP ES cell clones which suggests it arose late during the isolation of the *Stard7* targeted ES cell line, and is not found in the parental JM8N4 clone.

To further characterize the C57BL/6N-*Tyr^cWTSI^* line, we generated deep (∼45×) whole genome sequence of two albino founders; the genome of these mice being composed of C57BL/6N-Taconic and C57BL/6N ES cell-derived DNA. This analysis revealed only 549 homozygous private single nucleotide variants (SNV) in both sequenced mice that were not present in the reference C57BL/6J genome (Church *et al*., 2009; Waterston *et al.,* 2002), C57BL/6NJ, or in the genomes of 17 other laboratory mouse strains (Keane *et al.,* 2011; Wong *et al.,* 2012). Six hundred and forty six homozygous private indels, and 21 private structural variants (Table 1, Supporting Information Table 1) were also detected. We attempted to validate all 58 homozygous SNVs and 39 homozygous indel calls on Chr 7, which are linked to the *Tyr^cWTSI^* deletion, using the Sequenom MassARRAY® platform. A total of 41/43 SNVs and 7/7 homozygous indels were validated, with the remainder of sites failing in the oligo design phase due to the repetitive nature of the flanking sequence (Supporting Information Table 2). An estimated false-discovery rate of 1.3% for SNVs was derived by the validation of a further 155/157 homozygous SNVs randomly selected from the rest of the genome. Some heterozygous variants were also detected (Table 1), however, the high number of false positive SNV calls (51/131) and indel calls (11/17) indicate that the majority of the heterozygous calls are likely due to artefacts resulting from read mapping to the reference genome. To assess the origin of the variants present on chromosome 7 we genotyped DNA from the EPD0176_3_A10 ES cell clone, the parental JM8N4 line, and C57BL/6N-Taconic, and compared these genotypes to calls made in C57BL/6N-*Tyr^cWTSI^* (Supporting Information Table 2). This analysis revealed 31 variants (SNVs and indels) on chromosome 7 of C57BL/6N-*Tyr^cWTSI^* that are shared with both the EPD0176_3_A10 ES cell clone and the JM8N4 parental cell line, but not C57BL/6N-Taconic. Two of the SNVs originating from the JM8N4 parental cell line were predicted to cause amino acid changes (K/M at AA 107 of *Sult2a5* and G/E at AA 455 of *Zfp74*). A comparison of C57BL/6N-Taconic and C57BL/6N-*Tyr^cWTSI^* revealed that only 6 SNVs and one indel on chromosome 7 originate from C57BL/6N-Taconic. We were able to validate 8 SNVs which are unique to C57BL/6N-*Tyr^cWTSI^*. In addition to the *Tyr^cWTSI^* deletion, only one other validated structural variant unique to C57BL/6N-*Tyr^cWTSI^* was localized to chromosome 7; a 233bp homozygous insertion that fell within an intron of *Leng8*. Details of the variants detected by the initial genome sequencing and subsequent validation can be found in Supporting Information Data S1-S3, and Supporting Information Table 3.

**Table 1:** Comparison of the Genomes of the C57BL/6N-*Tyr^cWTS^*^*I*^ Founders and 18 Laboratory Mouse Strains

	Total number of C57BL/6N-*Tyr^cWTSI^* calls against the reference genome^a^	Total number of C57BL/6N-*Tyr^cWTSI^* calls shared with C57BL6/NJ^b^	Total number of C57BL/6N-*Tyr^cWTSI^* calls not found in the mouse genomes strains or FVB/NJ
SNVs Hom	10,897	10,261	549
Indels Hom	16,200	14,073	646
SNVs Het	36,497	31,623	3,965
Indels Het	64,323	31,132	6,309

a Includes only sites where SNV or indel positions were found in both C57BL/6N-*Tyr^cWTSI^* sequenced founder mice.

b Includes SNPs or indels in C57BL6/NJ from the mouse genomes project release^9^ and calls from C57BL6/NJ resequenced with 100bp Illumina paired end reads; indels were compared within a 25bp window.

Because variants not linked to chromosome 7 can be easily segregated away, further rounds of backcrossing to C57BL/6N-Taconic were performed prior to cryopreservation of the C57BL/6N-*Tyr^cWTSI^* strain. To validate the C57BL/6N-*Tyr^cWTSI^* line, *Tyr^cWTSI^* animals were test bred to chimeras from a new EUCOMM strain yielding black mice carrying the targeted ES derived allele while all albino offspring were wildtype (data not shown). Thus using the C57BL/6N-*Tyr^cWTSI^* strain germline transmission can be scored using coat color with only black chimera progeny requiring biopsying and genotyping.

## DISCUSSION

Although a large proportion of EUCOMM/KOMP clones carry the reconstructed agouti locus (Pettitt *et al.,* 2009) which can be used to select for potential GLT in certain conditions, the Sanger MGP uses albino C57BL/6-Tyr^c-Brd^ host blastocysts for microinjection (Liu *et al.,* 1998) which reduces its potential usefulness (this is also true for BALB/c blastocysts). Because of the dominant effect of agouti, the resulting breeding to nonagouti wildtype mice means that all mice may still have to be genotyped in order to detect GLT as a small percentage of nonagouti mice may also carry the targeted mutation. To test this we analyzed 181 Sanger MGP colonies produced from JM8 agouti cell lines; 25 colonies (13%) produced only black mice at G_1_ and would therefore have been missed using only agouti color selection. In addition, from a total of 1,351 G_1_ het mice detected by PCR, 712 (52%) had a black coat color; thus limiting the detection to just agouti mice may also have a significant effect on colony expansion rates in a high-throughput project where a rapid turnaround of GLT to phenotyping is at a premium.

Following backcrossing the C57BL/6N-*Tyr^cWTSI^* strain is almost identical to C57BL/6N-Taconic carrying just two variants that fall into coding sequence, 25 other single nucleotide variants on chromosome 7, and just one SV other than the *Tyr^cWTSI^* deletion. This strain will be of great utility for the International Mouse Phenotyping Consortium, which aims to generate and phenotype knockouts for all mouse protein-coding genes over the next decade. The spontaneous nature of the *Tyr^cWTSI^* mutation has advantages in that no foreign vector or *loxP* sequences are retained within the genome. The new colony is freely available to the research community.

## METHODS

### Mouse Production

The care and use of all mice in this study were in accordance with the UK Home Office regulations, UK Animals (Scientific Procedures) Act of 1986 and were approved by the Wellcome Trust Sanger Institute Ethical Review Committee. Embryonic stem cell (ESC) clone EPD0176_3_A10 from the EUCOMM resource was used to produce *Stard7^tm1a(EUCOMM)Wtsi^* mutant mice through standard blastocyst injection and chimera breeding techniques (Nagy *et al.,* 2003). C57BL/6-Tyr^c-Brd^ albino host blastocysts were used due to the ease of harvesting sufficient numbers and potential GLT of C57BL/6 over other strains (Schuster-Gossler *et al.,* 2001). Chimeras were bred to C57BL/6NTac females (Taconic) and offspring were genotyped by a qPCR assay that counts the number of neomycin phosphotransferase cassettes. NeoF GGTGGAGAGGCTATTCGGC; NeoR GAACACGGCGGCATCAG; NeoM1 TGGGCACAACAGACAATCGGCTG FAM. Cycling conditions are: x1 95°C 20 sec; x35 95°C 10 sec; 60°C 30 sec. G_1_ heterozygotes were backcrossed to C57BL/6NTac USA (Taconic) for one generation before heterozygous offspring were inter-crossed to obtain homozygous *Stard7^tm1a(EUCOMM)Wtsi^* mice. Two albino mice (male and female) arising from this inter-cross and not carrying the *Stard7^tm1a(EUCOMM)Wtsi^* allele were used to provide genomic DNA for the full genome sequencing experiments.

### Tiling Path Construction

A tiling path of 26 amplicons covering ∼24 kb with minimal overlap was designed using PCRTiler software. Reactions were performed on a selection of albino and wildtype control mice. Amplification conditions were 94°C 5 min, followed by 35 cycles of 94°C 30 sec, 58°C 30 sec, 72°C 1 min 30 sec, with a final extension of 72°C 5 min.

### Sequence Analysis

Illumina reads generated from whole genome sequencing were aligned to the mouse reference genome NCBIM37 with BWA version 0.5.9-r16 (Li and Durbin, 2009) and realignment around known indels was performed with the SAMtools calmd function [version 0.1.18-r572; (Li *et al.,* 2009)]. SNPs and indel discovery was performed with the SAMtools mpileup function and calling was performed with the BCFtools view function (Li, 2011). The vcf-annotate function in VCFtools package was used to filter the SNP and indel calls. To predict the functional consequences of SNP and indels we used the Variant Effect Predictor from Ensembl (McLaren *et al.,* 2010), and queried against Ensembl release 66 gene models. We compared the SNPs and indels from the albino mouse genomes to calls from the C57BL6/NJ genome, which was also sequenced with 100 bp read pairs, and to the SNPs and indels from the Mouse Genomes Project (Keane *et al.,* 2011) and the FVB/NJ genome (Wong *et al.,* 2012). Structural variants were called as described previously (Keane *et al.,* 2011), and compared to those identified in C57BL/6NJ, the Mouse Genomes Project, and FVB/NJ. Genotyping of SNPs and indels was performed using the Sequenom Mass iPLEX Gold Assay (Gabriel *et al.,* 2009).

Sequence data is available from the European Nucleotide Archive (ENA) under accession number ERP001554.

### Genotyping Protocol for C57BL/6N-Tyr^cWTSI^

The following primers were used to detect the *Tyr* deletion in heterozygotes. Tyr_MGP_F: GCTTCTTCATCCTGCTGGTC, Tyr_MGP_R: AAGCAGAGCAGGCTGACATT. Amplification conditions are 94°C 5 min, followed by 35 cycles of 94°C 30 sec, 58°C 30 sec, 72°C 45 sec, with a final extension of 72°C 5 min. A 169 bp product is observed in mutated animals.

## References

[b1] Bradley A, Anastassiadis K, Ayadi A, Battey JF, Bell C, Birling M-C, Bottomley J, Brown SD, Bürger A, Bult CJ, Bushell W, Collins FS, Desaintes C, Doe B, Economides A, Eppig JT, Finnell RH, Fletcher C, Fray M, Frendewey D, Friedel RH, Grosveld FG, Hansen J, Hérault Y, Hicks G, Hörlein A, Houghton R, Hrabé de Angelis M, Huylebroeck D, Iyer V, De Jong PJ, Kadin JA, Kaloff C, Kennedy K, Koutsourakis M, Kent Lloyd KC, Marschall S, Mason J, McKerlie C, McLeod MP, Von Melchner H, Moore M, Mujica AO, Nagy A, Nefedov M, Nutter LM, Pavlovic G, Peterson JL, Pollock J, Ramirez-Solis R, Rancourt DE, Raspa M, Remacle JE, Ringwald M, Rosen B, Rosenthal N, Rossant J, Ruiz Noppinger P, Ryder E, Schick JZ, Schnütgen F, Schofield P, Seisenberger C, Selloum M, Simpson EM, Skarnes WC, Smedley D, Stanford WL, Francis Stewart A, Stone K, Swan K, Tadepally H, Teboul L, Tocchini-Valentini GP, Valenzuela D, West AP, Yoshinaga Y, Wurst W (2012). The mammalian gene function resource: The international knockout mouse consortium. Mamm Genome.

[b2] Brown SDM, Moore MW (2012). The international mouse phenotyping consortium: Past and future perspectives on mouse phenotyping. Mamm Genome.

[b3] Church DM, Goodstadt L, Hillier LW, Zody MC, Goldstein S, She X, Bult CJ, Agarwala R, Cherry JL, DiCuccio M, Hlavina W, Kapustin Y, Meric P, Maglott D, Birtle Z, Marques AC, Graves T, Zhou S, Teague B, Potamousis K, Churas C, Place M, Herschleb J, Runnheim R, Forrest D, Amos-Landgraf J, Schwartz DC, Cheng Z, Lindblad-Toh K, Eichler EE, Ponting CP (2009). Lineage-specific biology revealed by a finished genome assembly of the mouse. PLoS Biol.

[b4] Gabriel S, Ziaugra L, Tabbaa D (2009). SNP genotyping using the sequenom massARRAY iPLEX platform. Curr Protocols Hum Genet.

[b5] Gervais AL, Marques M, Gaudreau L (2010). PCRTiler: Automated design of tiled and specific PCR primer pairs. Nucleic Acids Res.

[b6] Keane TM, Goodstadt L, Danecek P, White MA, Wong K, Yalcin B, Heger A, Agam A, Slater G, Goodson M, Furlotte NA, Eskin E, Nellåker C, Whitley H, Cleak J, Janowitz D, Hernandez-Pliego P, Edwards A, Belgard TG, Oliver PL, McIntyre RE, Bhomra A, Nicod J, Gan X, Yuan W, Van der Weyden L, Steward CA, Bala S, Stalker J, Mott R, Durbin R, Jackson IJ, Czechanski A, Guerra-Assunção JA, Donahue LR, Reinholdt LG, Payseur BA, Ponting CP, Birney E, Flint J, Adams DJ (2011). Mouse genomic variation and its effect on phenotypes and gene regulation. Nature.

[b7] Klüppel M, Beermann F, Ruppert S, Schmid E, Hummler E, Schütz G (1991). The mouse tyrosinase promoter is sufficient for expression in melanocytes and in the pigmented epithelium of the retina. Proc Natl Acad Sci USA.

[b8] Li H (2011). A statistical framework for SNP calling, mutation discovery, association mapping and population genetical parameter estimation from sequencing data. Bioinformatics.

[b9] Li H, Durbin R (2009). Fast and accurate short read alignment with Burrows-Wheeler transform. Bioinformatics.

[b10] Li H, Handsaker B, Wysoker A, Fennell T, Ruan J, Homer N, Marth G, Abecasis G, Durbin R (2009). The sequence alignment/map format and SAMtools. Bioinformatics.

[b11] Liu P, Zhang H, McLellan A, Vogel H, Bradley A (1998). Embryonic lethality and tumorigenesis caused by segmental aneuploidy on mouse chromosome 11. Genetics.

[b12] McLaren W, Pritchard B, Rios D, Chen Y, Flicek P, Cunningham F (2010). Deriving the consequences of genomic variants with the Ensembl API and SNP effect predictor. Bioinformatics.

[b13] Nagy A, Gertsenstein M, Vintersten K, Behringer R (2003). Manipulating the mouse embryo: A laboratory manual.

[b14] Pettitt SJ, Liang Q, Rairdan XY, Moran JL, Prosser HM, Beier DR, Lloyd KC, Bradley A, Skarnes WC (2009). Agouti C57BL/6N embryonic stem cells for mouse genetic resources. Nat Methods.

[b15] Schuster-Gossler K, Lee AW, Lerner CP, Parker HJ, Dyer VW, Scott VE, Gossler A, Conover JC (2001). Use of coisogenic host blastocysts for efficient establishment of germline chimeras with C57BL/6J ES cell lines. Biotechniques.

[b16] Skarnes WC, Rosen B, West AP, Koutsourakis M, Bushell W, Iyer V, Mujica AO, Thomas M, Harrow J, Cox T, Jackson D, Severin J, Biggs P, Fu J, Nefedov M, De Jong PJ, Stewart AF, Bradley A (2011). A conditional knockout resource for the genome-wide study of mouse gene function. Nature.

[b17] Waterston RH, Lindblad-Toh K, Birney E, Rogers J, Abril JF, Agarwal P, Agarwala R, Ainscough R, Alexandersson M, An P, Antonarakis SE, Attwood J, Baertsch R, Bailey J, Barlow K, Beck S, Berry E, Birren B, Bloom T, Bork P, Botcherby M, Bray N, Brent MR, Brown DG, Brown SD, Bult C, Burton J, Butler J, Campbell RD, Carninci P, Cawley S, Chiaromonte F, Chinwalla AT, Church DM, Clamp M, Clee C, Collins FS, Cook LL, Copley RR, Coulson A, Couronne O, Cuff J, Curwen V, Cutts T, Daly M, David R, Davies J, Delehaunty KD, Deri J, Dermitzakis ET, Dewey C, Dickens NJ, Diekhans M, Dodge S, Dubchak I, Dunn DM, Eddy SR, Elnitski L, Emes RD, Eswara P, Eyras E, Felsenfeld A, Fewell GA, Flicek P, Foley K, Frankel WN, Fulton LA, Fulton RS, Furey TS, Gage D, Gibbs RA, Glusman G, Gnerre S, Goldman N, Goodstadt L, Grafham D, Graves TA, Green ED, Gregory S, Guigó R, Guyer M, Hardison RC, Haussler D, Hayashizaki Y, Hillier LW, Hinrichs A, Hlavina W, Holzer T, Hsu F, Hua A, Hubbard T, Hunt A, Jackson I, Jaffe DB, Johnson LS, Jones M, Jones TA, Joy A, Kamal M, Karlsson EK, Karolchik D, Kasprzyk A, Kawai J, Keibler E, Kells C, Kent WJ, Kirby A, Kolbe DL, Korf I, Kucherlapati RS, Kulbokas EJ, Kulp D, Landers T, Leger JP, Leonard S, Letunic I, Levine R, Li J, Li M, Lloyd C, Lucas S, Ma B, Maglott DR, Mardis ER, Matthews L, Mauceli E, Mayer JH, McCarthy M, McCombie WR, McLaren S, McLay K, McPherson JD, Meldrim J, Meredith B, Mesirov JP, Miller W, Miner TL, Mongin E, Montgomery KT, Morgan M, Mott R, Mullikin JC, Muzny DM, Nash WE, Nelson JO, Nhan MN, Nicol R, Ning Z, Nusbaum C, Connor MJ, Okazaki Y, Oliver K, Overton-Larty E, Pachter L, Parra G, Pepin KH, Peterson J, Pevzner P, Plumb R, Pohl CS, Poliakov A, Ponce TC, Ponting CP, Potter S, Quail M, Reymond A, Roe BA, Roskin KM, Rubin EM, Rust AG, Santos R, Sapojnikov V, Schultz B, Schultz J, Schwartz MS, Schwartz S, Scott C, Seaman S, Searle S, Sharpe T, Sheridan A, Shownkeen R, Sims S, Singer JB, Slater G, Smit A, Smith DR, Spencer B, Stabenau A, Stange-Thomann N, Sugnet C, Suyama M, Tesler G, Thompson J, Torrents D, Trevaskis E, Tromp J, Ucla C, Ureta-Vidal A, Vinson JP, Von Niederhausern AC, Wade CM, Wall M, Weber RJ, Weiss RB, Wendl MC, West AP, Wetterstrand K, Wheeler R, Whelan S, Wierzbowski J, Willey D, Williams S, Wilson RK, Winter E, Worley KC, Wyman D, Yang S, Zdobnov EM, Zody MC, Lander ES (2002). Initial sequencing and comparative analysis of the mouse genome. Nature.

[b18] Wong K, Bumpstead S, Van Der Weyden L, Reinholdt LG, Wilming LG, Adams DJ, Keane TM (2012). Sequencing and characterization of the FVB/NJ mouse genome. Genome Biol.

