# Supplementary material for: Genomic Analysis of a Novel Spontaneous Albino C57BL/6N Mouse Strain
Source: Genesis. 2013 Apr 26;51(7):523–8. doi: 10.1002/dvg.22398 (PMC3799019; doi:10.1002/dvg.22398)
Supplement: Supplementary file 1 [file dvg0051-0523-sd1.rtf]

Supplementary Table 1: Structural variants called from the sequencing of C57BL/6N-TyrcWTSI founders with predicted NCBI m37 (mm9) sequence coordinates and PCR confirmation. For insertions, only the insertion site, and not the insertion size, was predicted.
SV	Chr	Start	End	Size	Type	Affected genes and zygoisity	PCR confirmation	
SV1	X	42967940	42967942	 	Insertion	homozygous 	 No product detected	
SV2	X	140030565	140030567	 	Insertion	Pak3 intron; homozygous	No product detected	
SV3	1	90609848	90615195	5347	Deletion	homozygous	yes but 361bp larger than predicted size	
SV4	1	182725311	182725960	649	Deletion	homozygous 	yes	
SV5	2	180357505	180357523	 1972	Insertion	tcfl5 intron	yes	
SV6	5	19083188	19083300	112	Deletion	Magi2 intron; homozygous	No product detected	
SV7	5	121252790	121252797	470 	Insertion	Oac1c intron; homozygous	Yes but as heterozygote	
SV8	6	34269510	34269512	 	Insertion	Intronic (predicted gene); heterozygous 	multiple bands and non-specific amplification	
SV9	7	4093253	4093270	 233	Insertion	Leng8 intron; homozygous	Yes	
SV10	7	94628580	94642881	14301	Deletion	Tyr 2 exons; homozygous	yes (albino mutation)	
SV11	8	79907482	79908006	524	Deletion	Arghgap10 intron; homozygous	yes	
SV12	10	117408578	117408598	 	Insertion	heterozygous 	No product detected	
SV13	12	94264334	94264439	105	Deletion	heterozygous	multiple bands and non-specific amplification	
SV14	13	54497353	54497355	 	Insertion	heterozygous 	No product detected	
SV15	14	81224081	81224083	 	Insertion	heterozygous	No product detected	
SV16	15	53540516	53540518	 285	Insertion	Samd12 intron; homozygous	yes	
SV17	16	51924290	51924292	 	Insertion	homozygous	No product detected	
SV18	17	94578293	94578307	 	Insertion	heterozygous	No product detected	
SV19	18	6240080	6240510	430	Deletion	intronic; homozygous in MDDH13.2g, heterozygous in MDDH13.2b	yes	
SV20	3	100432825	100439109	6284	Deletion	Man1a2; heterozygous in MDDH13.2b	yes	
SV21	19	42835007	42835009	 2800	Insertion	near Hps1 exon 42834990	only detected in WT littermate	

Supplementary Table 2: Sequenom confirmation of homozygous nucleotide variants called from chromosome 7 following the sequencing and analysis of two C57BL/6N-TyrcWTSI founders. Of the 41 homozygous SNVs assayed, 6 fall in protein-coding regions. All 6 indels and all 25 SNVs shared between C57BL/6N-TyrcWTSI  and the JM8N4 ES cell line are also shared with the EPD0176_3_A10 ESC clone, but not C57BL/6NTac. Similarly, the indel and 6 SNVs shared between C57BL/6N-TyrcWTSI  and C57BL/6NTac are not observed in either the JM8N4 ES cell line or the EPD0176_3_A10 ESC clone. The validated sites are listed in Supplementary Table 3.
Variant type	Number of assays 	True positive calla	C57BL/6N-TyrcWTSI onlyb	C57BL/6N-TyrcWTSI and JM8N4 parental cell linec	C57BL/6N-TyrcWTSI and  EPD0176_3_A10 ESC cloned	C57BL/6N-TyrcWTSI and C57BL/6N-Taconice	
Homozygous indel	7	7	0	6	6	1	
Homozygous SNV	41	39 
(+2 het.)	8	25	25	6	
Coding SNV	6	3	0	3	3	0	
a)	Sequence variants detected in C57BL/6N-TyrcWTSI were confirmed on the MassARRAY® platform (Sequenom)
b)	Sequence variants present in C57BL/6N-TyrcWTSI  but not in C57BL/6NTac USA (Taconic), JM8N4 ES cell line or the EPD0176_3_A10 ES cell clone; 2 SNV calls were genotyped by the MassARRAY platform as heterozygous in C57BL/6N-TyrcWTSI  only
c)	Sequence variants present in C57BL/6N-TyrcWTSI that are also present in the JM8N4 ESC line
d)	Sequence variants present in C57BL/6N-TyrcWTSI that are also present in the EPD0176_3_A10 ESC clone which produced the Tyr deletion 
e)	Sequence variants present in C57BL/6N-TyrcWTSI that are also present in the C57BL/6NTac USA (Taconic) strain used in the back-crossing and to expand the C57BL/6N-TyrcWTSI colony.
