# Supplementary material for: Genomic Analysis of a Novel Spontaneous Albino C57BL/6N Mouse Strain
Source: Genesis. 2013 Apr 26;51(7):523–8. doi: 10.1002/dvg.22398 (PMC3799019; doi:10.1002/dvg.22398)
Supplement: Supplementary file 5 [file dvg0051-0523-sd5.rtf]

##############################################
# Sanger Mouse Genetics Project              #
# Genomic Analysis of a Spontaneous          #
# Albino C57BL/6N strain                     #
# WELLCOME TRUST SANGER INSTITUTE            #
##############################################

# CONTACTS:

 Edward Ryder: er1@sanger.ac.uk
 Ramiro Ramirez-Solis: rrs@sanger.ac.uk

# This README and the corresponding files are supplementary data for the paper entitled:

 "Genomic analysis of a novel spontaneous albino C57BL/6N strain"

# Authors:

 Edward Ryder, Kim Wong, Diane Gleeson, Thomas M. Keane, Debarati
 Sethi, Sapna Vyas, Hannah Wardle-Jones, James N. Bussell, Richard
 Houghton, Jennifer Salisbury, Nina Harvey, David J. Adams, The
 Sanger Mouse Genetics Project and Ramiro Ramirez-Solis

# Files:

 C57BL6N_TyrcWTSI_hom_indels.vcf
 C57BL6N_TyrcWTSI_hom_snps.vcf

# Description:

 The files list high-confidence SNPs and indels called in both of the genomes of the C57BL/6N-TyrcWTSI founders (see Table 1), and absent from the 17 Mouse Genomes Project (Keane et al.) strains and FVB/NJ (Wong et al.). 

The columns labelled "MDDH13_2b" and "MDDH13_2g" represent the SNP or indel calls for the 2 founder mice.

 All SNPs and indels are relative to the reference genome C57BL/6J version NCBIM37 (mm9).

These files are in standard Variant Call Format (VCF):
http://www.1000genomes.org/wiki/Analysis/Variant%20Call%20Format/vcf-variant-call-format-version-41

 The SNP and indel consequences were annotated with Ensembl terms listed here:
 http://feb2012.archive.ensembl.org/info/docs/variation/predicted_data.html#consequences

 Gene models are from Ensembl version 66: http://feb2012.archive.ensembl.org/Mus_musculus/Info/Index


# References:

 Keane TM, Goodstadt L, Danecek P, White MA, et al. Mouse genomic variation and its effect on phenotypes and gene regulation. Nature. 2011 Sep 14;477(7364):289-94. doi: 10.1038/nature10413. PubMed PMID: 21921910; PubMed Central PMCID: PMC3276836

 Wong K, Bumpstead S, Van Der Weyden L, Reinholdt LG, Wilming LG, Adams DJ, Keane TM. Sequencing and characterization of the FVB/NJ mouse genome. Genome Biol. 2012 Aug 23;13(8):R72. [Epub ahead of print] PubMed PMID: 22916792; PubMed  Central PMCID: PMC3491372
